# Effectiveness of mass spectrometry and genomic analysis in the surveillance of nontuberculous *Mycobacterium* in Taiwan

**DOI:** 10.1128/jcm.00622-26

**Published:** 2026-08-14

**Authors:** Hsueh-Wei Chu, Hsin-Ni Huang, Chin-Chung Shu, Tai-Fen Lee, Yu-Tsung Huang

**Affiliations:** 1 Department of Laboratory Medicine, National Taiwan University Hospital 38006, Taipei, Taiwan; 2 Department of Internal Medicine, National Taiwan University Hospital 38006, Taipei, Taiwan; University of Western Australia, Perth, Australia

**Keywords:** whole-genome sequencing, MALDI-TOF MS, nontuberculous mycobacteria

## Abstract

Nontuberculous mycobacteria (NTM) are diverse, and species-level identification remains challenging in routine diagnostics. We analyzed NTM isolates collected at three regional centers of the National Taiwan University Hospital (NTUH) from 2019 to 2024 to assess geographic variation and identification performance after implementation of matrix-assisted laser desorption/ionization time-of-flight mass spectrometry (MALDI-TOF MS). Among 3,188 cases meeting the microbiological criteria for probable pulmonary NTM disease, the species distribution differed by region: *Mycobacterium avium* complex predominated in central Taiwan (Yunlin, 47.3%), whereas *M. abscessus* complex (Taipei, 26.5%) and *M. kansasii* (Hsinchu, 12.4%) were more common in northern Taiwan. In 2019, 14.5% of isolates were reported to be unidentified by MALDI-TOF MS; with workflow optimization and database updates, this percentage decreased but plateaued at 4.5–4.8%. Whole-genome sequencing (WGS) of 61 randomly selected persistently unidentified isolates revealed eight average nucleotide identity (ANI)-defined clusters; 55 isolates (90.2%) could not be assigned to known species using current reference databases. Two clusters detected only in Hsinchu were phylogenetically closest to *M. kyorinense*, with ANI values below the species demarcation threshold. Overall, we observed marked regional heterogeneity of NTM in Taiwan and a persistent identification gap that remained after MALDI-TOF MS optimization and follow-up WGS.

**IMPORTANCE:**

This study characterized regional differences in the NTM species distribution across Taiwan, and the results highlight the limitations of current identification approaches. MALDI-TOF MS identifies most isolates, but locally circulating lineages represent a persistent gap in global reference libraries. Even with whole-genome sequencing (WGS), 90.2% (55/61) of persistently unresolved isolates could not be assigned to known species in the current reference databases despite the formation of clear ANI- and phylogeny-defined clusters. These findings show that both proteomic and genomic reference resources for clinical NTM remain incomplete. Expanding regionally representative databases and performing WGS for isolates that remain unresolved by MALDI-TOF MS will be necessary to improve species-level resolution for surveillance and clinical interpretation.

## INTRODUCTION

Nontuberculous mycobacteria (NTM) comprising approximately 200 known species are ubiquitous in the environment. Their clinical impact varies significantly by species, ranging from localized skin and soft tissue infections to severe pulmonary and disseminated diseases, particularly in immunocompromised individuals (1, 2). In many developed countries, the incidence of NTM disease has been increasing in recent years (3, 4). This trend is attributed to an aging population, differences in immune status, and increasing comorbidities (5).

The diagnosis of NTM disease requires the integration of clinical, radiographic, and microbiologic criteria (6). Species-level identification informs treatment because drug susceptibility testing is clinically useful for only selected species–drug combinations, and therapeutic decisions often depend on the species involved (7, 8). Commercial nucleic acid-based assays can provide rapid identification, but coverage is incomplete, and a subset of isolates remain unassigned in routine practice (9). Matrix-assisted laser desorption ionization-time of flight mass spectrometry (MALDI-TOF MS) can identify a wider range of species with short turnaround times and low technical complexity. However, its performance depends on the representation of reference spectra, and locally circulating lineages may be underrepresented in global libraries, leading to identification failures (10).

NTM species distributions also vary geographically, which can affect both epidemiological interpretation and diagnostic performance. In Taiwan, regional patterns of NTM species have not been systematically assessed at the species level across multiple regions, and the genomic context of persistently unresolved isolates remains incompletely described. In this study, we analyzed NTM isolates collected from three regional centers between 2019 and 2024. We used MALDI-TOF MS for routine identification and applied whole-genome sequencing (WGS) to selected isolates that remained unresolved despite repeat processing and database updates. Our objectives were to characterize the regional NTM species distribution, evaluate the performance and limits of MALDI-TOF MS in a routine setting, and examine the genomic characteristics of unresolved isolates to bridge existing diagnostic gaps.

## MATERIALS AND METHODS

### Study design

We retrospectively analyzed NTM isolates identified by MALDI-TOF MS from all clinical specimens collected between 2019 and 2024 at three National Taiwan University Hospital (NTUH) branches in northern Taiwan (Taipei and Hsinchu) and central Taiwan (Yunlin).

A total of 21,084 NTM isolates were recovered during the study period, of which 19,501 were identified to the species level. These isolate-level data were used for laboratory-based analyses, including specimen distribution, unidentified rates, database-version comparisons, and selection of persistently unresolved isolates for whole-genome sequencing (WGS).

For epidemiologic analyses, isolates obtained from the same patient and assigned to the same species were counted as a single case. Probable NTM pulmonary disease (NTM-PD) was defined according to the ATS/IDSA microbiologic criteria as either two separate sputum cultures yielding the same species or one positive bronchial specimen (6). Using this case-based approach, 3,188 probable NTM-PD cases were identified and analyzed for species distribution and temporal trends. In addition, 315 extrapulmonary NTM isolates were identified descriptively and defined as recovery of NTM from any nonrespiratory specimen.

In February 2021, we implemented a quality improvement policy for routine NTM identification. We selected 99 isolates originally reported as unidentified to monitor this workflow and assess the impact of the MALDI-TOF MS database transition. These isolates underwent parallel retesting across database versions. With respect to the 61 isolates that remained unresolved after mass spectrometry reanalysis, we performed whole-genome sequencing (WGS) to determine their taxonomic classification and phylogenetic relationships.

### Clinical NTM isolation and MALDI-TOF MS identification protocol

Clinical specimens were inoculated into MGIT broth (BACTEC MGIT 960) and onto Löwenstein–Jensen medium (L–J medium) and incubated at 35°C in 5% CO₂. Acid-fast-positive isolates were subcultured on Middlebrook 7H11 agar for MALDI-TOF MS identification and stored at −80°C in Middlebrook 7H9 broth supplemented with 10% oleic acid–albumin–dextrose–catalase; corresponding laboratory records were retained.

For MALDI-TOF MS, colonies grown on Middlebrook 7H11 agar were processed using bead-beating extraction, followed by treatment with 70% formic acid (11). Spectra were matched against the MBT Mycobacteria databases, and a score of ≥1.70 was considered reliable for species-level identification (12). Routine identification was performed using the Microflex LT/SH System with *Mycobacterium* Library v4.0 (880 spectra) from April 2019 and the Sirius One System with *Mycobacterium* Library v7.0 (1,069 spectra) from November 2022. To minimize unidentified results, a quality improvement policy that included colony subculturing to reduce matrix interference and turbidity verification to ensure optimal protein extraction was implemented in February 2021. Although recent taxonomic revisions have proposed splitting the former genus *Mycobacterium* into multiple genera, the traditional genus name *Mycobacterium* remains widely used in clinical microbiology. For clarity and consistency with common clinical usage, we use the traditional nomenclature throughout this manuscript.

### Genomic DNA sequencing

Mycobacterial genomic DNA was extracted using the Presto gDNA Bacterial Advanced Kit (Geneaid) according to the manufacturer’s protocol. The purity of the extracted genomic DNA was assessed using a NanoDrop 1000 v3.8 spectrophotometer (Thermo Fisher Scientific, Massachusetts, USA). DNA quantification was performed using a Qubit 3.0 fluorometer with a dsDNA High-Sensitivity (HS) Assay Kit (Invitrogen, Carlsbad, USA), while the DNA fragment size distribution was characterized via a Qsep100 Capillary Electrophoresis System (BiOptic, New Taipei City, Taiwan). For library preparation, 1 μg of gDNA was used to construct paired-end libraries with a TruSeq DNA PCR-Free Library Prep Kit (Illumina, San Diego, USA) following the manufacturer’s instructions. Samples were indexed using TruSeq DNA Unique Dual (UD) Indexes v2 (Illumina, San Diego, USA). After library pooling, whole-genome sequencing was executed on the MiniSeq platform using a mid-output (MO) flow cell (300 cycles, 2 × 150 bp) with a final loading concentration of 4.5 pM.

### Bioinformatics analysis of the sequencing data

Raw sequencing reads underwent quality trimming before *de novo* assembly using Unicycler (13), with a subsequent assembly quality assessment performed via SeqFu (14). To determine genomic relatedness, the average nucleotide identity (ANI) between isolates was calculated using FastANI (15). Taxonomic assignment was then conducted using the Genome Taxonomy Database Toolkit (GTDB-Tk), which classifies isolate genomes on the basis of ANI via comparison against organisms in the GTDB reference database (16).

Phylogenetic relationships were determined by pangenome analysis. The finalized genomes were annotated with Prokka (17), and the core genes—defined as those present in at least 80% of the strains, including both unidentified isolates and NCBI type strains—were identified and aligned to assess protein sequence similarity using Roary (18). A maximum likelihood phylogenetic tree was subsequently constructed using RAxML (19) and visualized with MEGA-X (20). Additionally, potential virulence genes were identified by screening the WGS data against the Virulence Factor Database (VFDB) using VFanalyzer (21).

## RESULTS

### Specimen distribution of the data set

**Fig 1:**
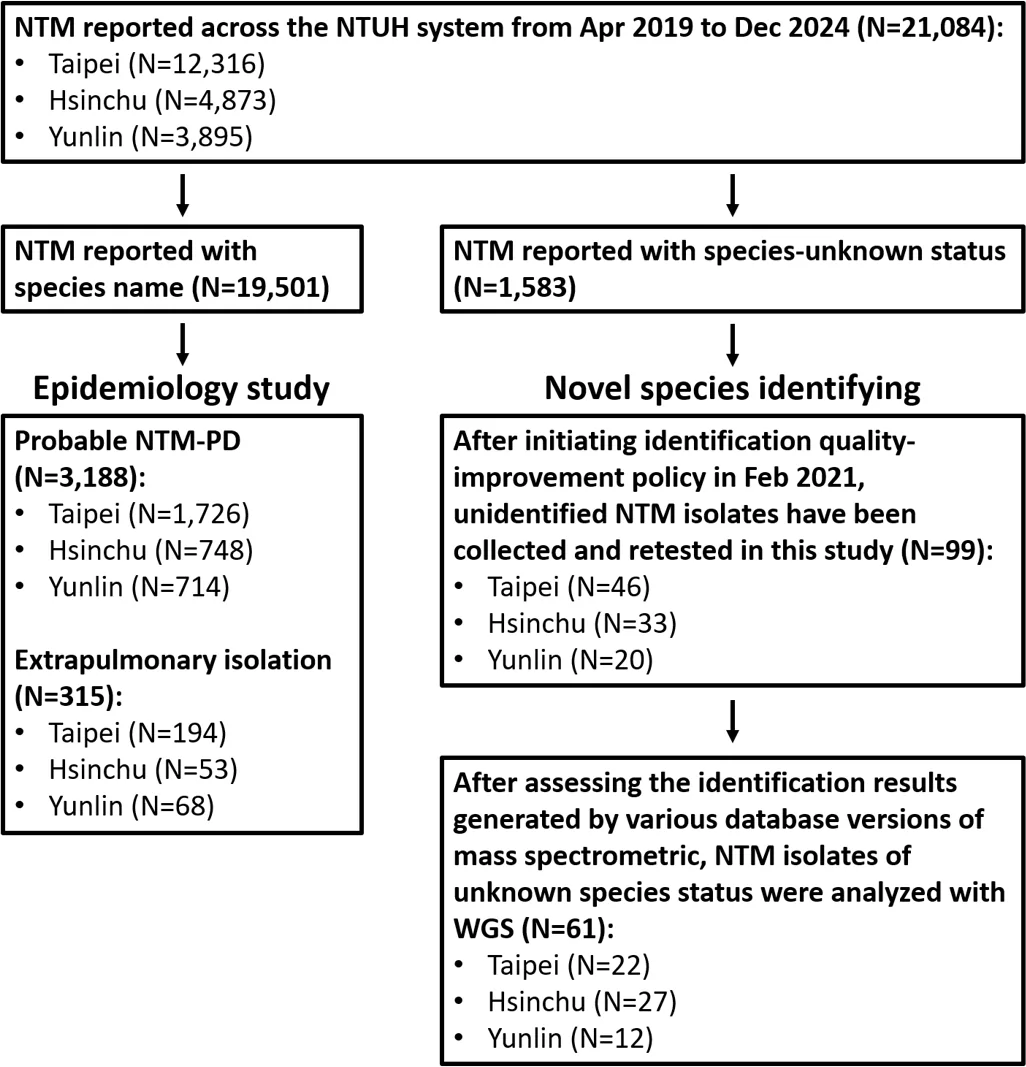
Study design for NTM epidemiology surveillance in Taiwan. NTM-PD, NTM pulmonary disease; WGS, whole-genome sequencing.

During the study period, a total of 21,084 NTM isolates were reported across the NTUH system, comprising 12,316 from Taipei, 4,873 from Hsinchu (northern Taiwan), and 3,895 from Yunlin (central Taiwan) (Fig. 1). Among these NTM isolates, 20,095 (95.3%) were recovered from respiratory specimens, including sputum (*N* = 19,337), bronchial wash or lavage (*N* = 656), and lung biopsy samples (*N* = 102). The remaining 989 NTM isolates (4.7%) were obtained from extrapulmonary specimens, including skin and soft tissue specimens (*N* = 603), peripheral blood (*N* = 106), sterile site specimens (*N* = 66), bone marrow (*N* = 4), and other sources (*N* = 210).

### Performance of MALDI-TOF MS databases

The Bruker *Mycobacterium* Library update from v4.0 to v7.0 added 23 new species and expanded the reference spectra for 44 existing species (Table S1). In the matched 6-month periods before and after the update, the proportion of *Mycobacterium intracellulare* increased from 22.6% to 26.0% (*P* = 0.0367), and *M. timonense* was detected only after the update (0% vs 0.4%, *P* = 0.0027), whereas the *M. colombiense* proportion decreased from 1.8% to 0.9% (*P* = 0.0118) (Table 1). In contrast, the proportion of *M. mageritense* decreased from 5.6% to 3.2% (*P* = 0.0002). Major groups, such as the *M. avium* complex (MAC) and the *M. abscessus* complex, changed little across versions, suggesting that the database update affected some species-level calls but did not substantially alter the overall epidemiologic pattern.

**TABLE 1:** Comparison of NTM identification results in matched 6-month periods before and after the Bruker Mycobacterium Library update from v4.0 to v7.0^*a*^

NTM identification results	v4.0 (*N* = 2066)		v7.0 (*N* = 2044)		Total (*N* = 4110)	*P* value^*b*^
	*N*	%	*N*	%		
*Mycobacterium avium* complex	735	35.6%	757	37.0%	1492	0.5690
*Mycobacterium intracellulare*	466	22.6%	532	26.0%	998	0.0367
*Mycobacterium avium*	187	9.1%	158	7.7%	345	0.1185
*Mycobacterium marseillense*	40	1.9%	35	1.7%	75	0.5637
*Mycobacterium colombiense*	38	1.8%	19	0.9%	57	0.0118
*Mycobacterium timonense*		0.0%	9	0.4%	9	0.0027
*Mycobacterium mantenii*	3	0.1%	4	0.2%	7	0.7055
Unspecific species in *Mycobacterium avium* complex	1	<0.1%		0.0%	1	–
*Mycobacterium abscessus* complex	513	24.8%	510	25.0%	1023	0.9253
*Mycobacterium fortuitum*	175	8.5%	172	8.4%	347	0.8721
*Mycobacterium kansasii*	113	5.5%	130	6.4%	243	0.2755
*Mycobacterium paragordonae*	115	5.6%	109	5.3%	224	0.6885
*Mycobacterium mageritense*	116	5.6%	65	3.2%	181	0.0002
*Mycobacterium gordonae*	55	2.7%	73	3.6%	128	0.1116
*Mycobacterium chelonae*	65	3.1%	54	2.6%	119	0.3133
*Mycobacterium simiae* complex	34	1.6%	41	2.0%	75	0.4189
*Mycobacterium lentiflavum*	14	0.7%	17	0.8%	31	0.5900
*Mycobacterium simiae*	5	0.2%	5	0.2%	10	1.0000
*Mycobacterium interjectum*	4	0.2%	5	0.2%	9	0.7389
*Mycobacterium kubicae*	3	0.1%	5	0.2%	8	0.4795
*Mycobacterium paraense*	4	0.2%	3	0.1%	7	0.7055
*Mycobacterium parascrofulaceum*	1	<0.1%	6	0.3%	7	0.0588
*Mycobacterium sherrisii*	3	0.1%		0.0%	3	0.0833
*Mycobacterium mucogenicum/phocaicum*	33	1.6%	19	0.9%	52	0.0522
*Mycobacterium porcinum*	18	0.9%	17	0.8%	35	0.8658
*Mycobacterium senegalense*	17	0.8%	15	0.7%	32	0.7237
*Mycobacterium szulgai*	6	0.3%	12	0.6%	18	0.1573
*Mycobacterium seoulense*	4	0.2%	8	0.4%	12	0.2482
*Mycobacterium canariasense/cosmeticum*		0.0%	12	0.6%	12	0.0005
*Mycobacterium cosmeticum*	11	0.5%		0.0%	11	0.0009
*Mycobacterium algericus*	5	0.2%	5	0.2%	10	1.0000
*Mycobacterium peregrinum*	3	0.1%	7	0.3%	10	0.2059
*Mycobacterium kumamotonensis*	5	0.2%	2	0.1%	7	0.2568
*Mycobacterium neoaurum*	6	0.3%	1	<0.1%	7	0.0588
*Mycobacterium arupensis*	3	0.1%	3	0.1%	6	1.0000
*Mycobacterium farcinogenes*	4	0.2%	1	<0.1%	5	0.1797
*Mycobacterium scrofulaceum*	1	<0.1%	4	0.2%	5	0.1797
*Mycobacterium brisbanense*	3	0.1%	1	<0.1%	4	0.3173
*Mycobacterium nebraskense*	1	<0.1%	3	0.1%	4	0.3173
*Mycobacterium immunogenum*	1	<0.1%	2	0.1%	3	0.5637
*Mycobacterium nonchromogenicus*	2	0.1%	1	<0.1%	3	0.5637
*Mycobacterium boenickei*	1	<0.1%	2	0.1%	3	0.5637
*Mycobacterium septicum*	1	<0.1%	2	0.1%	3	0.5637
*Mycobacterium canariasense*	3	0.1%		0.0%	3	0.0833
*Mycobacterium terrae*	3	0.1%		0.0%	3	0.0833
*Mycobacterium gastri*	1	<0.1%	1	<0.1%	2	–
*Mycobacterium novocastrense*	1	<0.1%	1	<0.1%	2	–
*Mycobacterium xenopi*	1	<0.1%	1	<0.1%	2	–
*Mycobacterium iranicum*	2	0.1%		0.0%	2	–
*Mycobacterium wolinskyi*	2	0.1%		0.0%	2	–
*Mycobacterium angelicum*		0.0%	2	0.1%	2	–
*Mycobacterium fortuitum*		0.0%	2	0.1%	2	–
*Mycobacterium obuense*		0.0%	2	0.1%	2	–
*Mycobacterium asiaticum*	1	<0.1%		0.0%	1	–
*Mycobacterium bacteremicum*	1	<0.1%		0.0%	1	–
*Mycobacterium conceptionense*	1	<0.1%		0.0%	1	–
*Mycobacterium kyorinense*	1	<0.1%		0.0%	1	–
*Mycobacterium neworleansense*	1	<0.1%		0.0%	1	–
*Mycobacterium paraffinicum*	1	<0.1%		0.0%	1	–
*Mycobacterium salmoniphilum*	1	<0.1%		0.0%	1	–
*Mycobacterium arcueilense*		0.0%	1	<0.1%	1	–
*Mycobacterium goodii*		0.0%	1	<0.1%	1	–
*Mycobacterium haemophilum*		0.0%	1	<0.1%	1	–
*Mycobacterium heraklionensis*		0.0%	1	<0.1%	1	–
*Mycobacterium marinum*		0.0%	1	<0.1%	1	–
*Mycobacterium minnesotensis*		0.0%	1	<0.1%	1	–
*Mycobacterium phlei*		0.0%	1	<0.1%	1	–
Total	2066	100.0%	2044	100.0%	4110	

^*a*^ Values are shown as numbers and percentages of isolates assigned to each taxon. Differences in the overall distribution of identification results between the two periods were assessed by the *χ* test based on isolate counts.

^*b*^ –, statistical significance was not assessed because the sample size was too small to calculate a *P* value.

### Pulmonary NTM disease across three centers

**Fig 2:**
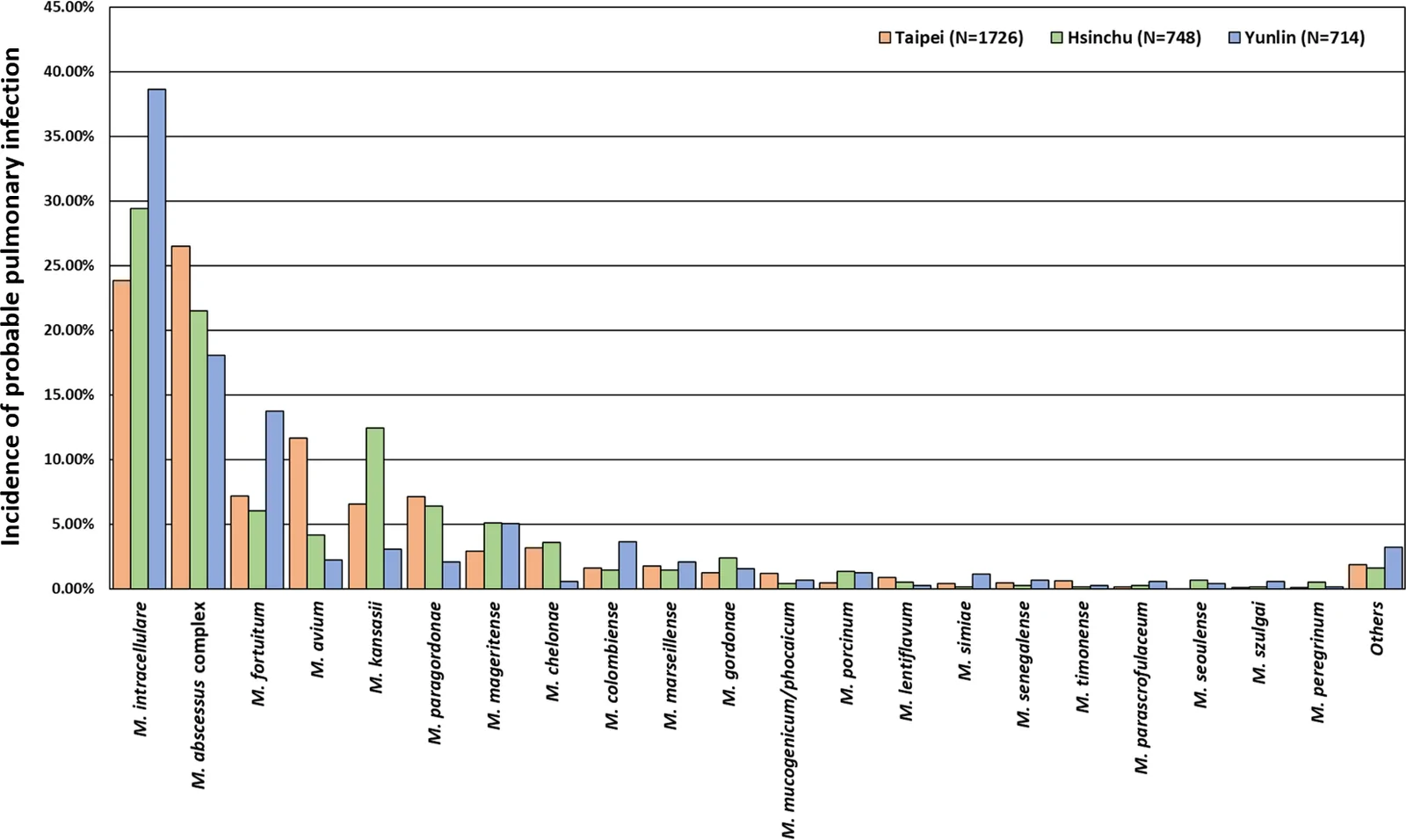
Distribution of major species causing probable pulmonary NTM disease across the three NTUH centers.

To investigate the epidemiology of probable NTM pulmonary disease (NTM-PD) in the data set, 3,188 cases were included from Taipei (*N* = 1,726), Hsinchu (*N* = 748), and Yunlin (*N* = 714), and the five most frequently identified species in NTM-PD were *M. intracellulare* (28.5%), *M. abscessus* complex (23.5%), *M. fortuitum* (8.4%), *M. avium* (7.8%), and *M. kansasii* (7.2%). Geographical variation in the most common species was observed. The top five species in Taipei were *M. abscessus* complex (26.5%), *M. intracellulare* (23.9%), *M. avium* (11.7%), *M. fortuitum* (7.3%), and *M. paragordonae* (7.1%). In Hsinchu, the top five species were *M. intracellulare* (29.4%), *M. abscessus* complex (21.5%), *M. kansasii* (12.4%), *M. paragordonae* (6.4%), and *M. fortuitum* (6.0%). Finally, in Yunlin, the top five species were *M. intracellulare* (38.7%), *M. abscessus* complex (18.1%), *M. fortuitum* (13.7%), *M. mageritense* (5.0%), and *M. colombiense* (3.6%) (Fig. 2).

MAC was the most prevalent species group nationwide (*N* = 1,296), and its prevalence exhibited geographic variation, with a higher proportion observed in central Taiwan than in northern Taiwan (47.3% in Yunlin vs 39.6% in Taipei and 36.8% in Hsinchu) (Table 2). *M. intracellulare* was more prevalent in Yunlin (38.7%) than in Taipei (23.9%) or Hsinchu (29.4%), whereas *M. avium* was more frequent in Taipei (11.7%) than in Hsinchu (4.1%) or Yunlin (2.2%). Other slow-growing species also showed regional variation. *M. kansasii* was more common in Hsinchu (12.4%) than in Taipei (6.6%) or Yunlin (3.1%), whereas *M. simiae* complex was isolated more frequently in Taipei (1.6%) and Yunlin (2.5%) than in Hsinchu (1.1%).

**TABLE 2:** Distribution of probable pulmonary NTM disease across the three NTUH centers^*a*^

NTM identification results	Incidence of probable pulmonary infection							
	Taipei (*N* = 1726)		Hsinchu (*N* = 748)		Yunlin (*N* = 714)		Total (*N* = 3188)	
	*N*	%	*N*	%	*N*	%	*N*	%
*Mycobacterium avium* complex	683	39.6%	275	36.8%	338	47.3%	1296	40.7%
*Mycobacterium intracellulare*	412	23.9%	220	29.4%	276	38.7%	908	28.5%
*Mycobacterium avium*	201	11.7%	31	4.1%	16	2.2%	248	7.8%
*Mycobacterium colombiense*	28	1.6%	11	1.5%	26	3.6%	65	2.0%
*Mycobacterium marseillense*	31	1.8%	11	1.5%	15	2.1%	57	1.8%
*Mycobacterium timonense*	11	0.6%	1	0.1%	2	0.3%	14	0.4%
*Mycobacterium mantenii*		0.0%	1	0.1%	2	0.3%	3	0.1%
Unspecific species in *Mycobacterium avium* complex		0.0%		0.0%	1	0.1%	1	<0.1%
*Mycobacterium abscessus* complex	458	26.5%	161	21.5%	129	18.1%	748	23.5%
*Mycobacterium fortuitum*	126	7.3%	45	6.0%	98	13.7%	267	8.4%
*Mycobacterium kansasii*	113	6.6%	93	12.4%	22	3.1%	228	7.2%
*Mycobacterium paragordonae*	123	7.1%	48	6.4%	15	2.1%	186	5.8%
*Mycobacterium mageritense*	50	2.9%	38	5.1%	36	5.0%	124	3.9%
*Mycobacterium chelonae*	55	3.2%	27	3.6%	4	0.6%	86	2.7%
*Mycobacterium simiae* complex	28	1.6%	8	1.1%	18	2.5%	54	1.7%
*Mycobacterium lentiflavum*	15	0.9%	4	0.5%	2	0.3%	21	0.7%
*Mycobacterium simiae*	7	0.4%	1	0.1%	8	1.1%	16	0.5%
*Mycobacterium parascrofulaceum*	3	0.2%	2	0.3%	4	0.6%	9	0.3%
*Mycobacterium interjectum*		0.0%	1	0.1%	2	0.3%	3	0.1%
*Mycobacterium kubicae*	1	0.1%		0.0%	1	0.1%	2	0.1%
*Mycobacterium paraense*	1	0.1%		0.0%		0.0%	1	<0.1%
*Mycobacterium sherrisii*	1	0.1%		0.0%		0.0%	1	<0.1%
*Mycobacterium europaeum*		0.0%		0.0%	1	0.1%	1	<0.1%
*Mycobacterium gordonae*	22	1.3%	18	2.4%	11	1.5%	51	1.6%
*Mycobacterium mucogenicum/phocaicum*	21	1.2%	3	0.4%	5	0.7%	29	0.9%
*Mycobacterium porcinum*	8	0.5%	10	1.3%	9	1.3%	27	0.8%
*Mycobacterium senegalense*	8	0.5%	2	0.3%	5	0.7%	15	0.5%
*Mycobacterium seoulense*		0.0%	5	0.7%	3	0.4%	8	0.3%
*Mycobacterium szulgai*	2	0.1%	1	0.1%	4	0.6%	7	0.2%
*Mycobacterium peregrinum*	2	0.1%	4	0.5%	1	0.1%	7	0.2%
*Mycobacterium kyorinense*	4	0.2%		0.0%	1	0.1%	5	0.2%
*Mycobacterium neoaurum*	3	0.2%	1	0.1%	1	0.1%	5	0.2%
*Mycobacterium brisbanense*	4	0.2%		0.0%	1	0.1%	5	0.2%
*Mycobacterium xenopi*	1	0.1%	2	0.3%	1	0.1%	4	0.1%
*Mycobacterium asiaticum*	1	0.1%	1	0.1%	1	0.1%	3	0.1%
*Mycobacterium kumamotonensis*	2	0.1%	1	0.1%		0.0%	3	0.1%
*Mycobacterium canariasense/cosmeticum*	1	0.1%		0.0%	2	0.3%	3	0.1%
*Mycobacterium farcinogenes*		0.0%		0.0%	3	0.4%	3	0.1%
*Mycobacterium algericus*		0.0%	1	0.1%	1	0.1%	2	0.1%
*Mycobacterium cosmeticum*	2	0.1%		0.0%		0.0%	2	0.1%
*Mycobacterium novocastrense*	1	0.1%		0.0%	1	0.1%	2	0.1%
*Mycobacterium wolinskyi*		0.0%	1	0.1%	1	0.1%	2	0.1%
*Mycobacterium goodii*	1	0.1%		0.0%	1	0.1%	2	0.1%
*Mycobacterium paraffinicum*	1	0.1%		0.0%		0.0%	1	<0.1%
*Mycobacterium shinjukuense*		0.0%	1	0.1%		0.0%	1	<0.1%
*Mycobacterium immunogenum*	1	0.1%		0.0%		0.0%	1	<0.1%
*Mycobacterium koreensis*		0.0%	1	0.1%		0.0%	1	<0.1%
*Mycobacterium heraklionensis*	1	0.1%		0.0%		0.0%	1	<0.1%
*Mycobacterium virginiensis*		0.0%		0.0%	1	0.1%	1	<0.1%
*Mycobacterium longobardus*	1	0.1%		0.0%		0.0%	1	<0.1%
*Mycobacterium septicum*	1	0.1%		0.0%		0.0%	1	<0.1%
*Mycobacterium canariasense*	1	0.1%		0.0%		0.0%	1	<0.1%
*Mycobacterium obuense*	1	0.1%		0.0%		0.0%	1	<0.1%
*Mycobacterium boenickei*		0.0%		0.0%	1	0.1%	1	<0.1%
*Mycobacterium conceptionense*		0.0%	1	0.1%		0.0%	1	<0.1%

^*a*^ Numbers and percentages are shown for each species in Taipei, Hsinchu, and Yunlin.

Among rapid-growing species, *M. abscessus* complex, the most frequently isolated pathogen, was more prevalent in Taipei (26.5%) and Hsinchu (21.5%) than in Yunlin (18.1%). Conversely, *M. fortuitum* was notably more prevalent in Yunlin (13.7%) than in Taipei (7.3%) or Hsinchu (6.0%).

### Extrapulmonary NTM across three centers

A total of 315 extrapulmonary NTM isolates were identified from Taipei (*N* = 194), Hsinchu (*N* = 53), and Yunlin (*N* = 68), with species distributions varying markedly by specimen type (Table S2). *M. abscessus* complex was the predominant species in sterile site specimens, including pleural fluid (33.3%), ascites (75.0%), synovial fluid (75.0%), and cerebrospinal fluid (100.0%). This species also predominated in nonsterile or localized colonization samples, such as wounds and pus (52.0%), drainage (47.4%), and gastrointestinal–hepatobiliary samples (57.1%). In peripheral blood, *M. avium* (29.7%) and *M. abscessus* complex (20.3%) were most frequently recovered, whereas *M. abscessus* complex (33.3%), *M. colombiense* (33.3%), and *M. kansasii* (33.3%) were isolated at the same rate from bone marrow isolates. In contrast, urine and stool samples exhibited more diverse species compositions.

### Evaluating the unidentified results of MALDI-TOF MS and database upgrades

**Fig 3:**
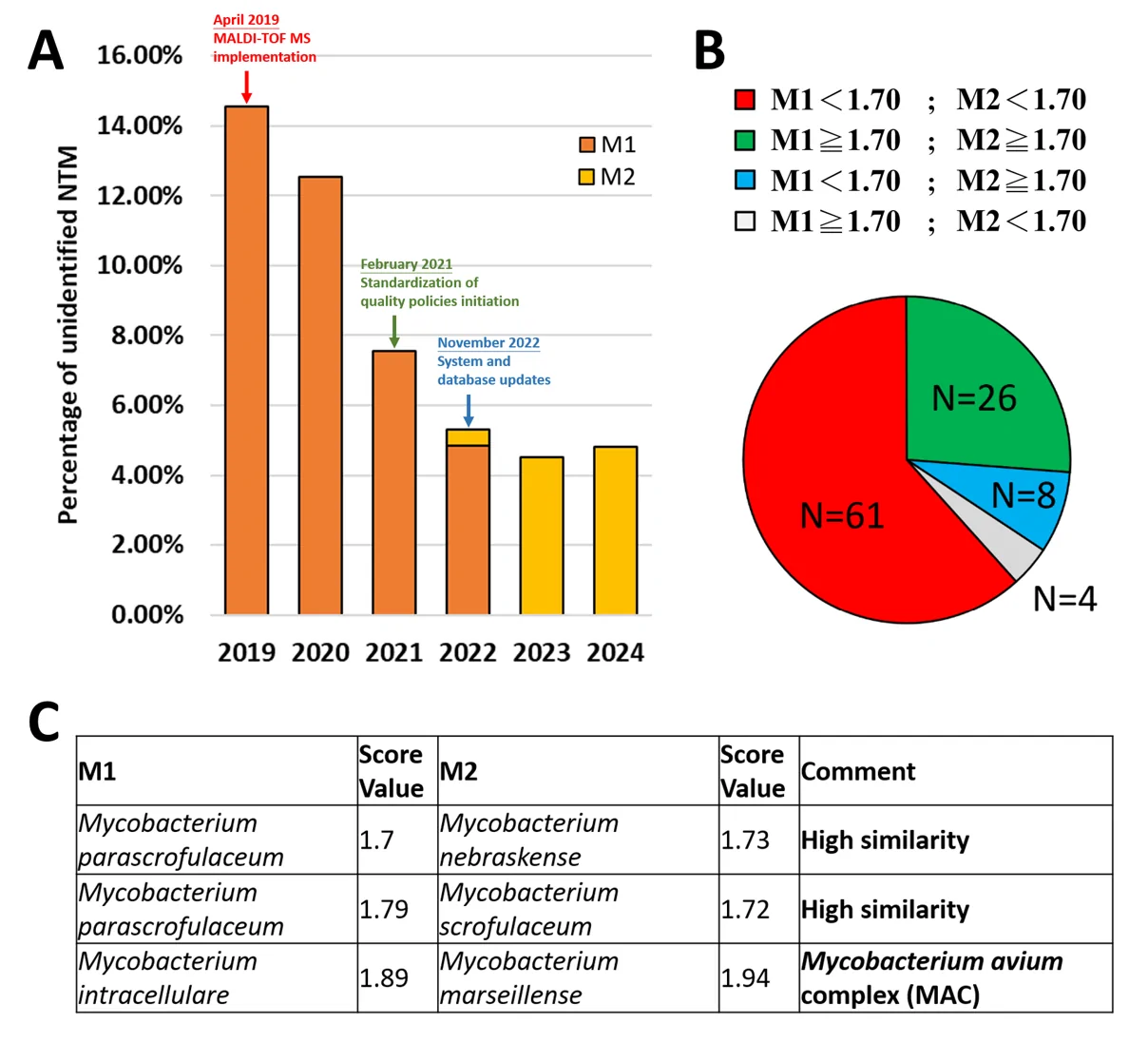
MALDI-TOF MS identification performance before and after workflow optimization and database transition. (**A**) Annual proportion of NTM isolates reported as unidentified between 2019 and 2024. (**B**) Parallel reanalysis of 99 isolates originally reported as unidentified using two Bruker MALDI-TOF MS platforms. (**C**) Discordant identification results observed between the two platforms. M1, Bruker Biotyper Microflex LT/SH with Mycobacterium Library v4.0; M2, Bruker Biotyper Sirius One with Mycobacterium Library v7.0.

Overall, 1,583 of 21,084 isolates (7.5%) were reported as unidentified during the study period. The initial identification failure rate was 14.5% following the implementation of MALDI-TOF MS in April 2019, but the failure rate gradually declined to 7.6% in 2021 when a quality improvement policy was initiated within the clinical laboratory. However, despite subsequent system and software upgrades in November 2022, the rate of unidentified isolates plateaued at 4.5%–4.8% (Fig. 3A).

To evaluate the effectiveness of the quality improvement policy in reducing the identification failure rate, continuous monitoring was implemented. A total of 99 NTM isolates, which had remained unidentified since February 2021, were reanalyzed using both the Microflex and Sirius One systems and the Mycobacterial Library v4.0 and v7.0 databases. Among these NTM isolates, 26.3% (*N* = 26) fulfilled the criteria for successful identification (Fig. 3B). A few identification results differed between the two systems for three isolates (*N* = 3); however, these discrepancies had a negligible impact on clinical and epidemiological interpretation. This was because *M. scrofulaceum* and *M. parascrofulaceum* share high similarity and both exhibit low local prevalence (*N* = 2), while the remaining isolate consistently clustered within the MAC (*N* = 1) (Fig. 3C). Furthermore, while 8.1% (*N* = 8) of the isolates reached acceptable identification scores exclusively with the updated system and database, 4.0% (*N* = 4) fulfilled the criteria for the v4.0 database but not for v7.0. The remaining 61.6% of the isolates (*N* = 61) could not be identified to the species level by either system. This indicates that the hardware upgrade and database expansion provided only limited improvement in our setting. It also suggests that some locally circulating lineages are still underrepresented in current reference libraries.

### Assessment of WGS for clinical unidentified NTM isolates

To determine the taxonomic status of the 61 NTM isolates that remained unidentified via MALDI-TOF MS, whole-genome sequencing (WGS) of isolates from Taipei (*N* = 22), Hsinchu (*N* = 27), and Yunlin (*N* = 12) was performed. The Q30 percentage for all of the strains was above 85%, with N50 values ranging between 10^5^ and 10^6^ bp. Taxonomic assignment was conducted by calculating the ANI against the strains in the GTDB database. The analysis revealed that only 9.8% (*N* = 6) of these isolates could be assigned to known species, specifically *M. ahvazicum* (*N* = 1), *M. arosiense* (*N* = 1), *M. barrassiae* (*N* = 1), *M. sinensis* (*N* = 2), and *M. heraklionensis* (*N* = 1) (Table S3). Conversely, a significant majority of the isolates (90.2%, *N* = 55) remained unassigned to any established species; this group comprised 36 isolates with ANI scores below the 95% threshold and 19 isolates that yielded no matches within the database. These findings indicate that current reference databases could not assign most of these isolates from Taiwan to known species.

**Fig 4:**
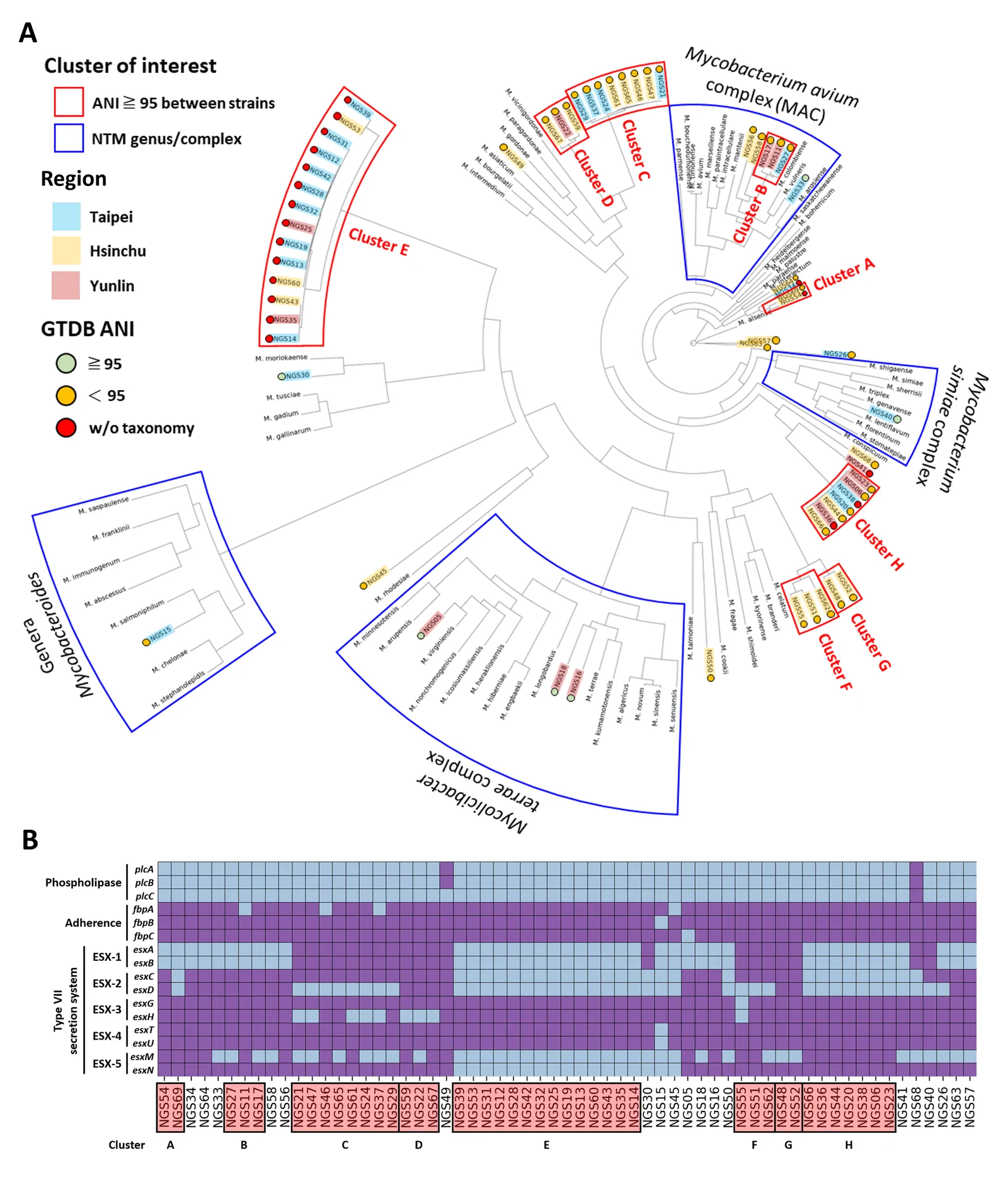
Whole-genome sequencing of persistently unresolved NTM isolates. (**A**) Maximum-likelihood phylogenetic tree based on core-genome analysis of the unresolved NTM isolates and selected reference strains. (**B**) Gene distribution profile based on screening by WGS against the VFDB. Genes related to phospholipase, adherence, and the Type VII secretion system are shown. The strains are arranged in the same order as in panel **A**. Purple indicates the presence of homologous open reading frames, and blue indicates no detected match.

To determine whether specific NTM strains recur within clinical settings, we evaluated their phylogenetic relationships and genomic divergence through pan-genome analysis. By analyzing core genes shared by at least 80% of the strains, 1,729 loci were identified and utilized for downstream analysis. A maximum-likelihood phylogenetic tree revealed only one cluster aligned with the MAC, and WGS analysis validated a total of eight clusters, within which the intracluster ANI exceeded 95% (Fig. 4A). None of the isolates within these eight clusters could reliably be assigned to a species with currently available databases, suggesting that these clusters represent previously uncharacterized or newly emerging NTM species native to Taiwan.

To evaluate the pathogenic potential of these emerging NTM clusters—specifically, whether they are predisposed to colonization or active infection—we systematically screened for mycobacterium-related virulence factors. Previous reports have implicated adherence factors, phospholipase C, and the Type VII secretion system (T7SS) as key determinants of NTM pathogenesis, and the results demonstrated that clusters C, D, F, and G harbored nearly all the substrate types associated with the T7SS, whereas the others encoded only a few virulence genes (Fig. 4B).

Five isolates in clusters F and G that were isolated exclusively from Hsinchu and identified as being most closely related to *M. kyorinense* via ANI analysis (90.9%–91.3% similarity) exhibited distinct regiospecific characteristics. These strains appeared as acid-fast, slow-growing, scotochromogen colonies on L–J medium from 30°C to 35°C. These findings suggest that these novel NTM isolates possess a repertoire of virulence factors that may facilitate their emergence as clinically significant pathogens.

## DISCUSSION

In this study, we systematically assessed regional variations in NTM species distributions across Taiwan. By differentiating members within the MAC, we revealed that *M. avium* was predominant in Taipei (an urban center), whereas *M. intracellulare* was more prevalent in Yunlin, a predominantly rural county. To our knowledge, such species-level distributions have not been extensively investigated in Taiwan primarily because the clinical implementation of NTM identification remains limited (22). Studies in Australia and Korea have identified *M. intracellulare* as the primary cause of NTM pulmonary disease, whereas *M. avium* remains predominant in Japan (23–25). Overall, these results suggest that species-level differentiation is essential for rigorous epidemiological surveillance, facilitating a more comprehensive understanding of the geographical diversity of NTM.

Our finding that *M. abscessus* complex is predominant in northern Taiwan is consistent with the results of several prior multicenter studies (22, 26). Nevertheless, the clinical utility of MALDI-TOF MS is limited by its failure to differentiate the three subspecies within the *M. abscessus* complex. Consequently, methods, such as Fourier transform infrared (FT-IR) spectroscopy or other advanced molecular diagnostic assays, are needed for accurate subspecies-level identification (27, 28). Such precision is essential for effective clinical management, as macrolide resistance patterns differ significantly across these subspecies (29).

The Bruker *Mycobacterium* Library for MALDI-TOF MS has undergone several updates, and previous studies have shown improved species-level identification after database expansion (30). In our data set, however, the transition from v4.0 to v7.0 had only a modest effect. The largest change in any single species was for *M. intracellulare* (3.4%), which was still smaller than the regional differences across centers. The v7.0 library did allow identification of additional MAC species, such as *M. timonense*, although the practical impact was limited by their low prevalence in our data set. Some of the apparent changes were related to differences in database reporting rather than true shifts in species distribution because v7.0 merged *M. canariasense* with *M. cosmeticum* and *M. austroafricanum* with *M. vanbaalenii* into single taxa (Table S1). Overall, the benefit of the update for initially unresolved isolates was limited, as only a small proportion could be newly assigned and the majority remained unidentified at the species level. These findings indicate that the current library still does not fully reflect the NTM diversity encountered in clinical practice.

Various sample processing protocols have been developed to obtain high-quality mass spectra from NTM (31). In our institution, challenges in spectral acquisition emerged immediately after adoption of the bead-beating protocol. These issues were likely related to interference from specimen matrix components during spectrum acquisition and the difficulty of standardizing biomass input because rapidly and slowly growing NTM species require different cell densities for optimal detection. Previous studies have also highlighted quality concerns related to the waxy cell wall of *M. abscessus* complex, the use of an insufficient ethanol drying time, and variation in optimal cultivation duration (32). To mitigate the suboptimal identification results, we implemented a standardized laboratory policy in which isolates with a score below the acceptable threshold underwent colony subculture to reduce matrix interference before reanalysis, followed by protein denaturation with 70% formic acid and mandatory turbidity verification to ensure an adequate protein concentration. Notably, while Rodríguez-Temporal et al. reported identification failure rates as high as 12.3% using similar MS-based platforms, our quality improvement initiative was associated with a marked reduction in the failure rate from 14.5% to 4.5–4.8% (33). This suggests that our optimized workflow achieves a higher identification success rate, supporting the improved reliability of MALDI-TOF MS-based NTM identification in our clinical laboratory workflow.

WGS has revealed genomic diversity among NTM isolates from clinical and environmental sources. In our data set, five isolates from Hsinchu formed two clusters most closely related to *M. kyorinense*, but their ANI values remained below the 95% threshold for species delineation. This pattern suggests that these isolates are related to but distinct from the currently recognized *M. kyorinense*. The available laboratory records were limited, but in one patient with possible NTM-PD, two respiratory isolates yielded low-confidence MALDI-TOF MS results, with *M. kyorinense* reported as the top match. Published reports of *M. kyorinense* infection remain limited and have described pulmonary disease mainly in patients with underlying cavitary lung lesions (34, 35). The prevalence and clinical relevance of these *M. kyorinense*-related lineages in northern Taiwan remain unclear.

Our study has several limitations. First, the WGS analysis was based on short-read sequencing, which did not allow complete assembly of the unresolved isolates. We therefore interpreted the ANI results together with the results of the phylogenetic analysis when assessing their relatedness to known species, including *M. kyorinense*-related lineages. Second, our parallel comparison was limited to isolates that had already been reported as unidentified. As a result, we did not systematically assess how often the two versions of the MALDI-TOF MS database might generate species-level identifications but disagree with each other in routine clinical isolates. Third, the 3,188 cases of probable NTM-PD included in the study were defined mainly by microbiologic criteria, and complete clinical and radiographic data were not available for all patients. The true prevalence of NTM-PD may, therefore, have been overestimated. Finally, although the study involved three regional centers, all the isolates were collected within a single medical system, which may limit the generalizability of the findings. Broader multicenter studies will be needed to confirm these results and better define the epidemiology of unresolved NTM lineages.

In conclusion, our study demonstrates marked regional variation in NTM species distributions across Taiwan, highlighting the importance of species-level differentiation for accurate epidemiologic surveillance. Although MALDI-TOF MS remains a practical tool for routine NTM identification and workflow optimization reduced the identification failure rate, a substantial proportion of isolates remained unresolved, even after database upgrades and subsequent WGS-based analysis against current reference databases. These findings suggest that local NTM diversity is incompletely represented in existing commercial and genomic reference resources and support the continued expansion of regionally informed databases and integrated genomic surveillance in clinical microbiology practice.

## Data Availability

The NTM data in this study are available upon reasonable request from the corresponding author, Y.-T.H., and the WGS data have been deposited in the NCBI archive under project accession number PRJNA1481133.

## SUPPLEMENTAL MATERIAL

Supplemental tables and figure jcm.00622-26-s0001.docx Tables S1 and S2; Fig. S1.

ASM does not own the copyrights to Supplemental Material that may be linked to, or accessed through, an article. The authors have granted ASM a non-exclusive, world-wide license to publish the Supplemental Material files. Please contact the corresponding author directly for reuse.
