# Supplementary material for: Effectiveness of mass spectrometry and genomic analysis in the surveillance of nontuberculous Mycobacterium in Taiwan
Source: J Clin Microbiol. 2026 Aug 14;64(9):e00622-26. doi: 10.1128/jcm.00622-26 (PMC13556190; doi:10.1128/jcm.00622-26)
Supplement: Supplementary file 1 [file jcm.00622-26-s0001.docx]

Supplementary Table 1. Differences between Bruker Mycobacterium Library v4.0 and v7.0. The table lists species with expanded reference spectra in v7.0 and species that were newly added to the database. Taxa treated as indistinguishable groups in v7.0 are indicated in the footnotes.

| Spectrum comparison between Mycobacterium Library v4.0 and v7.0 | | |
| --- | --- | --- |
| Spectrum expansion (N=44) | | New species (N=23) |
| *M. abscessus* complex | *M. koreense* | *M. alsense* |
| *M. agri* | *M. kubicae* | *M. angelicum* |
| *M. arosiense* | *M. mageritense* | *M. anyangense* |
| *M. arupense* | *M. mantenii* | *M. aquaticum* |
| *M. asiaticum* | *M. mucogenicum*** | *M. arcueilense* |
| *M. aurum* | *M. nebraskense* | *M. basiliense* |
| *M. austroafricanum** | *M. neoaurum* | *M. eburneum* |
| *M. canariasense* | *M. nonchromogenicum* | *M. grossiae* |
| *M. chelonae* | *M. noviomagense* | *M. helvum* |
| *M. cosmeticum* | *M. palustre* | *M. icosiumassiliensis* |
| *M. intracellulare* | *M. paragordonae* | *M. lehmannii* |
| *M. elephantis* | *M. phocaicum*** | *M. lutetiense* |
| *M. engbaekii* | *M. salmoniphilum* | *M. montmartrense* |
| *M. europaeum* | *M. septicum* | *M. neumannii* |
| *M. farcinogenes* | *M. shimoidei* | *M. paraterrae* |
| *M. fluoranthenivorans* | *M. stomatepiae* | *M. saopaulense* |
| *M. franklinii* | *M. szulgai* | *M. sarraceniae* |
| *M. goodii* | *M. thermoresistibile* | *M. saskatchewanense* |
| *M. heckeshornense* | *M. vaccae* | *M. shigaense* |
| *M. heidelbergense* | *M. vanbaalenii** | *M. stephanolepidis* |
| *M. hippocampi* | *M. wolinskyi* | *M. talmoniae* |
| *M. interjectum* |  | *M. timonense* |
| *M. iranicum* |  | *M. virginiense* |
| **M. austroafricanum* and *M. vanbaalenii* were in an indistinguishable taxonomic group in v7.0.  ***M. canariasense* and *M. cosmeticum* were in an indistinguishable taxonomic group in v7.0. | | |

Supplementary Table 2. Distribution of extrapulmonary NTM isolation across the three NTUH centers. The numbers and percentages are shown for each species in Taipei, Hsinchu, and Yunlin. Stool isolates are shown descriptively only and were not used to define probable NTM disease. At our institution, stool mycobacterial culture may be performed in selected patients, such as HIV-infected patients with persistent diarrhea, as part of the clinical evaluation for possible MAC involvement.

| NTM identification results | Extrapulmonary NTM isolates by specimen type | | | | | | | |
| --- | --- | --- | --- | --- | --- | --- | --- | --- |
|  | Taipei (N=194) | | Hsinchu (N=53) | | Yunlin (N=68) | | Total | |
|  | N | % | N | % | N | % | N | % |
| Stool | 52 |  | 14 |  | 12 |  | 78 |  |
| *Mycobacterium intracellulare* | 7 | 13.46% | 4 | 28.57% | 3 | 25.00% | 14 | 17.95% |
| *Mycobacterium avium* | 9 | 17.31% | 2 | 14.29% | 2 | 16.67% | 13 | 16.67% |
| *Mycobacterium fortuitum* | 8 | 15.38% | 1 | 7.14% | 2 | 16.67% | 11 | 14.10% |
| *Mycobacterium kansasii* | 3 | 5.77% | 3 | 21.43% | 3 | 25.00% | 9 | 11.54% |
| *Mycobacterium abscessus* complex | 6 | 11.54% |  | 0.00% |  | 0.00% | 6 | 7.69% |
| *Mycobacterium gordonae* | 4 | 7.69% | 1 | 7.14% |  | 0.00% | 5 | 6.41% |
| *Mycobacterium paragordonae* | 4 | 7.69% |  | 0.00% |  | 0.00% | 4 | 5.13% |
| *Mycobacterium marseillense* | 3 | 5.77% |  | 0.00% |  | 0.00% | 3 | 3.85% |
| *Mycobacterium chelonae* | 3 | 5.77% |  | 0.00% |  | 0.00% | 3 | 3.85% |
| *Mycobacterium parascrofulaceum* | 1 | 1.92% | 1 | 7.14% |  | 0.00% | 2 | 2.56% |
| *Mycobacterium mageritense* | 1 | 1.92% |  | 0.00% | 1 | 8.33% | 2 | 2.56% |
| *Mycobacterium colombiense* |  | 0.00% | 1 | 7.14% | 1 | 8.33% | 2 | 2.56% |
| *Mycobacterium seoulense* | 1 | 1.92% |  | 0.00% |  | 0.00% | 1 | 1.28% |
| *Mycobacterium mucogenicum/phocaicum* | 1 | 1.92% |  | 0.00% |  | 0.00% | 1 | 1.28% |
| *Mycobacterium porcinum* | 1 | 1.92% |  | 0.00% |  | 0.00% | 1 | 1.28% |
| *Mycobacterium arupensis* |  | 0.00% | 1 | 7.14% |  | 0.00% | 1 | 1.28% |
| Wound/Pus | 48 |  | 6 |  | 21 |  | 75 |  |
| *Mycobacterium abscessus* complex | 28 | 58.33% | 1 | 16.67% | 10 | 47.62% | 39 | 52.00% |
| *Mycobacterium fortuitum* | 7 | 14.58% |  | 0.00% | 3 | 14.29% | 10 | 13.33% |
| *Mycobacterium chelonae* | 5 | 10.42% |  | 0.00% |  | 0.00% | 5 | 6.67% |
| *Mycobacterium colombiense* | 1 | 2.08% | 1 | 16.67% | 2 | 9.52% | 4 | 5.33% |
| *Mycobacterium marinum* | 2 | 4.17% | 1 | 16.67% |  | 0.00% | 3 | 4.00% |
| *Mycobacterium avium* | 2 | 4.17% |  | 0.00% | 1 | 4.76% | 3 | 4.00% |
| *Mycobacterium intracellulare* |  | 0.00% | 2 | 33.33% | 1 | 4.76% | 3 | 4.00% |
| *Mycobacterium marseillense* | 1 | 2.08% |  | 0.00% | 1 | 4.76% | 2 | 2.67% |
| *Mycobacterium senegalense* | 1 | 2.08% |  | 0.00% | 1 | 4.76% | 2 | 2.67% |
| *Mycobacterium scrofulaceum* | 1 | 2.08% |  | 0.00% |  | 0.00% | 1 | 1.33% |
| *Mycobacterium thermoresistibile* |  | 0.00% | 1 | 16.67% |  | 0.00% | 1 | 1.33% |
| *Mycobacterium canariasense* |  | 0.00% |  | 0.00% | 1 | 4.76% | 1 | 1.33% |
| *Mycobacterium mageritense* |  | 0.00% |  | 0.00% | 1 | 4.76% | 1 | 1.33% |
| Peripheral blood | 36 |  | 10 |  | 18 |  | 64 |  |
| *Mycobacterium avium* | 12 | 33.33% | 3 | 30.00% | 4 | 22.22% | 19 | 29.69% |
| *Mycobacterium abscessus* complex | 7 | 19.44% | 4 | 40.00% | 2 | 11.11% | 13 | 20.31% |
| *Mycobacterium kansasii* | 5 | 13.89% |  | 0.00% | 3 | 16.67% | 8 | 12.50% |
| *Mycobacterium intracellulare* | 4 | 11.11% | 1 | 10.00% | 3 | 16.67% | 8 | 12.50% |
| *Mycobacterium haemophilum* | 3 | 8.33% |  | 0.00% |  | 0.00% | 3 | 4.69% |
| *Mycobacterium colombiense* | 1 | 2.78% | 1 | 10.00% | 1 | 5.56% | 3 | 4.69% |
| *Mycobacterium fortuitum* | 1 | 2.78% |  | 0.00% | 2 | 11.11% | 3 | 4.69% |
| *Mycobacterium mageritense* | 1 | 2.78% |  | 0.00% | 2 | 11.11% | 3 | 4.69% |
| *Mycobacterium parascrofulaceum* | 1 | 2.78% |  | 0.00% |  | 0.00% | 1 | 1.56% |
| *Mycobacterium cosmeticum* | 1 | 2.78% |  | 0.00% |  | 0.00% | 1 | 1.56% |
| *Mycobacterium porcinum* |  | 0.00% | 1 | 10.00% |  | 0.00% | 1 | 1.56% |
| *Mycobacterium marseillense* |  | 0.00% |  | 0.00% | 1 | 5.56% | 1 | 1.56% |
| Pleural fluid | 16 |  | 5 |  | 6 |  | 27 |  |
| *Mycobacterium abscessus* complex | 8 | 50.00% |  | 0.00% | 1 | 16.67% | 9 | 33.33% |
| *Mycobacterium intracellulare* | 4 | 25.00% | 1 | 20.00% | 2 | 33.33% | 7 | 25.93% |
| *Mycobacterium haemophilum* | 1 | 6.25% | 1 | 20.00% |  | 0.00% | 2 | 7.41% |
| *Mycobacterium kansasii* |  | 0.00% | 1 | 20.00% | 1 | 16.67% | 2 | 7.41% |
| *Mycobacterium avium* | 1 | 6.25% |  | 0.00% |  | 0.00% | 1 | 3.70% |
| *Mycobacterium gastri* | 1 | 6.25% |  | 0.00% |  | 0.00% | 1 | 3.70% |
| *Mycobacterium neoaurum* | 1 | 6.25% |  | 0.00% |  | 0.00% | 1 | 3.70% |
| *Mycobacterium mageritense* |  | 0.00% | 1 | 20.00% |  | 0.00% | 1 | 3.70% |
| *Mycobacterium mucogenicum/phocaicum* |  | 0.00% | 1 | 20.00% |  | 0.00% | 1 | 3.70% |
| *Mycobacterium fortuitum* |  | 0.00% |  | 0.00% | 1 | 16.67% | 1 | 3.70% |
| *Mycobacterium peregrinum* |  | 0.00% |  | 0.00% | 1 | 16.67% | 1 | 3.70% |
| Urine | 19 |  | 11 |  | 2 |  | 32 |  |
| *Mycobacterium gordonae* | 3 | 15.79% | 4 | 36.36% |  | 0.00% | 7 | 21.88% |
| *Mycobacterium chelonae* | 4 | 21.05% | 1 | 9.09% |  | 0.00% | 5 | 15.63% |
| *Mycobacterium abscessus* complex | 3 | 15.79% |  | 0.00% |  | 0.00% | 3 | 9.38% |
| *Mycobacterium paragordonae* | 2 | 10.53% | 1 | 9.09% |  | 0.00% | 3 | 9.38% |
| *Mycobacterium avium* | 1 | 5.26% | 1 | 9.09% |  | 0.00% | 2 | 6.25% |
| *Mycobacterium kansasii* | 1 | 5.26% | 1 | 9.09% |  | 0.00% | 2 | 6.25% |
| *Mycobacterium lentiflavum* | 1 | 5.26% | 1 | 9.09% |  | 0.00% | 2 | 6.25% |
| *Mycobacterium fortuitum* |  | 0.00% | 1 | 9.09% | 1 | 50.00% | 2 | 6.25% |
| *Mycobacterium intracellulare* | 1 | 5.26% |  | 0.00% |  | 0.00% | 1 | 3.13% |
| *Mycobacterium szulgai* | 1 | 5.26% |  | 0.00% |  | 0.00% | 1 | 3.13% |
| *Mycobacterium trivialis* | 1 | 5.26% |  | 0.00% |  | 0.00% | 1 | 3.13% |
| *Mycobacterium nonchromogenicus* | 1 | 5.26% |  | 0.00% |  | 0.00% | 1 | 3.13% |
| *Mycobacterium porcinum* |  | 0.00% | 1 | 9.09% |  | 0.00% | 1 | 3.13% |
| *Mycobacterium marseillense* |  | 0.00% |  | 0.00% | 1 | 50.00% | 1 | 3.13% |
| Drainage | 12 |  | 2 |  | 5 |  | 19 |  |
| *Mycobacterium abscessus* complex | 7 | 58.33% | 1 | 50.00% | 1 | 20.00% | 9 | 47.37% |
| *Mycobacterium intracellulare* | 2 | 16.67% |  | 0.00% | 1 | 20.00% | 3 | 15.79% |
| *Mycobacterium fortuitum* | 1 | 8.33% |  | 0.00% | 1 | 20.00% | 2 | 10.53% |
| *Mycobacterium kansasii* | 1 | 8.33% |  | 0.00% |  | 0.00% | 1 | 5.26% |
| *Mycobacterium sherrisii* | 1 | 8.33% |  | 0.00% |  | 0.00% | 1 | 5.26% |
| *Mycobacterium marinum* |  | 0.00% | 1 | 50.00% |  | 0.00% | 1 | 5.26% |
| *Mycobacterium stephanolepidis* |  | 0.00% |  | 0.00% | 1 | 20.00% | 1 | 5.26% |
| *Mycobacterium porcinum* |  | 0.00% |  | 0.00% | 1 | 20.00% | 1 | 5.26% |
| Gastrointestinal-hepatobiliary | 5 |  | 0 |  | 2 |  | 7 |  |
| *Mycobacterium abscessus* complex | 3 | 60.00% |  |  | 1 | 50.00% | 4 | 57.14% |
| *Mycobacterium kansasii* | 1 | 20.00% |  |  |  | 0.00% | 1 | 14.29% |
| *Mycobacterium fortuitum* | 1 | 20.00% |  |  |  | 0.00% | 1 | 14.29% |
| *Mycobacterium chelonae* |  | 0.00% |  |  | 1 | 50.00% | 1 | 14.29% |
| Ascites | 2 |  | 2 |  | 0 |  | 4 |  |
| *Mycobacterium abscessus* complex | 2 | 100.00% | 1 | 50.00% |  |  | 3 | 75.00% |
| *Mycobacterium avium* |  | 0.00% | 1 | 50.00% |  |  | 1 | 25.00% |
| Bone morrow | 0 |  | 1 |  | 2 |  | 3 |  |
| *Mycobacterium colombiense* |  |  | 1 | 100.00% |  | 0.00% | 1 | 33.33% |
| *Mycobacterium abscessus* complex |  |  |  | 0.00% | 1 | 50.00% | 1 | 33.33% |
| *Mycobacterium kansasii* |  |  |  | 0.00% | 1 | 50.00% | 1 | 33.33% |
| Synovial fluid | 2 |  | 2 |  | 0 |  | 4 |  |
| *Mycobacterium abscessus* complex | 1 | 50.00% | 2 | 100.00% |  |  | 3 | 75.00% |
| *Mycobacterium marinum* | 1 | 50.00% |  | 0.00% |  |  | 1 | 25.00% |
| CSF | 2 |  | 0 |  | 0 |  | 2 |  |
| *Mycobacterium abscessus* complex | 2 | 100.00% |  |  |  |  | 2 | 100.00% |

Supplementary Table 3. Characteristics of unidentified NTM with WGS analysis.

| **#Strain** | **#Region** | **#Cluster** | **Specimen** | **Closest classification of ANI** | **ANI** | **%Q30** | **N50** |
| --- | --- | --- | --- | --- | --- | --- | --- |
| NGS54 | H | A | sputum | N/A | - | 93.49% | 693146 |
| NGS69 | H | A | sputum | *Mycobacterium paraense* | 87.59 | 92.61% | 677519 |
| NGS34 | T | - | sputum | N/A | - | 91.18% | 464702 |
| NGS64 | H | - | sputum | *Mycobacterium paraense* | 87.49 | 91.70% | 869738 |
| NGS33 | T | - | sputum | *Mycobacterium arosiense* | 99.31 | 90.93% | 232461 |
| NGS27 | T | B | sputum | *Mycobacterium senriense* | 89.81 | 92.10% | 574986 |
| NGS11 | Y | B | sputum | *Mycobacterium senriense* | 89.72 | 85.64% | 177640 |
| NGS17 | Y | B | sputum | *Mycobacterium colombiense* | 89.63 | 92.01% | 492748 |
| NGS58 | H | - | sputum | *Mycobacterium colombiense* | 89.55 | 93.40% | 165607 |
| NGS56 | H | - | sputum | *Mycobacterium colombiense* | 88.88 | 93.38% | 365523 |
| NGS21 | T | C | sputum | *Mycobacterium kiyosense* | 82.88 | 92.62% | 366447 |
| NGS47 | H | C | sputum | *Mycobacterium kiyosense* | 83.05 | 90.31% | 180273 |
| NGS46 | H | C | sputum | *Mycobacterium kiyosense* | 82.98 | 90.42% | 174122 |
| NGS65 | H | C | sputum | *Mycobacterium kiyosense* | 82.92 | 92.50% | 223652 |
| NGS61 | H | C | sputum | *Mycobacterium kiyosense* | 83.01 | 92.29% | 345913 |
| NGS24 | T | C | sputum | *Mycobacterium kiyosense* | 82.85 | 92.87% | 241630 |
| NGS37 | T | C | sputum | *Mycobacterium kiyosense* | 82.93 | 91.45% | 201527 |
| NGS29 | T | C | sputum | *Mycobacterium kiyosense* | 82.88 | 92.56% | 211277 |
| NGS59 | H | D | sputum | *Mycobacterium kiyosense* | 83.05 | 93.06% | 190616 |
| NGS22 | Y | D | sputum | *Mycobacterium kiyosense* | 83.33 | 91.26% | 195423 |
| NGS67 | H | D | sputum | *Mycobacterium kiyosense* | 83.17 | 92.23% | 184014 |
| NGS49 | H | - | sputum | *Mycobacterium paragordonae* | 85.51 | 89.53% | 195331 |
| NGS39 | T | E | sputum | N/A | - | 91.46% | 277745 |
| NGS53 | H | E | sputum | N/A | - | 93.47% | 264857 |
| NGS31 | T | E | sputum | N/A | - | 91.08% | 364994 |
| NGS12 | T | E | sputum | N/A | - | 85.92% | 328577 |
| NGS42 | T | E | sputum | N/A | - | 90.55% | 329337 |
| NGS28 | T | E | sputum | N/A | - | 92.70% | 553876 |
| NGS32 | T | E | sputum | N/A | - | 91.23% | 388959 |
| NGS25 | Y | E | sputum | N/A | - | 91.92% | 476676 |
| NGS19 | T | E | sputum | N/A | - | 92.55% | 668870 |
| NGS13 | T | E | sputum | N/A | - | 86.02% | 329345 |
| NGS60 | H | E | sputum | N/A | - | 93.63% | 474101 |
| NGS43 | H | E | sputum | N/A | - | 90.35% | 474033 |
| NGS35 | Y | E | sputum | N/A | - | 91.27% | 267398 |
| NGS14 | T | E | sputum | N/A | - | 85.65% | 228759 |
| NGS30 | T | - | sputum | *Mycobacterium barrassiae* | 99.18 | 92.59% | 183567 |
| NGS15 | T | - | sputum | *Mycobacterium salmoniphilum* | 91.07 | 91.63% | 654617 |
| NGS45 | H | - | sputum | *Mycobacterium chitae* | 81.76 | 90.47% | 185206 |
| NGS05 | Y | - | sputum | *Mycobacterium heraklionensis* | 98.89 | 86.11% | 124179 |
| NGS18 | Y | - | sputum | *Mycobacterium sinensis* | 98.15 | 92.55% | 438977 |
| NGS16 | Y | - | sputum | *Mycobacterium sinensis* | 99.6 | 92.28% | 393418 |
| NGS50 | H | - | sputum | *Mycobacterium talmoniae* | 84.41 | 90.61% | 127117 |
| NGS55 | H | F | sputum | *Mycobacterium kyorinense* | 91.34 | 92.29% | 173384 |
| NGS51 | H | F | sputum | *Mycobacterium kyorinense* | 91.33 | 92.92% | 231935 |
| NGS62 | H | F | sputum | *Mycobacterium kyorinense* | 91.03 | 91.96% | 471406 |
| NGS48 | H | G | sputum | *Mycobacterium kyorinense* | 90.92 | 90.68% | 402923 |
| NGS52 | H | G | sputum | *Mycobacterium kyorinense* | 90.91 | 92.99% | 277881 |
| NGS66 | H | H | sputum | *Mycobacterium malmoense* | 81.32 | 92.20% | 260786 |
| NGS36 | Y | H | sputum | N/A | - | 91.16% | 260810 |
| NGS44 | H | H | sputum | *Mycobacterium malmoense* | 81.35 | 90.31% | 245086 |
| NGS20 | T | H | sputum | *Mycobacterium heidelbergense* | 81.28 | 92.65% | 111777 |
| NGS38 | T | H | sputum | N/A | - | 90.39% | 234468 |
| NGS06 | Y | H | sputum | *Mycobacterium malmoense* | 81.41 | 85.69% | 167974 |
| NGS23 | Y | H | sputum | *Mycobacterium malmoense* | 81.43 | 92.71% | 263808 |
| NGS41 | Y | - | sputum | N/A | - | 90.31% | 144200 |
| NGS68 | H | - | sputum | *Mycobacterium palustre* | 81.85 | 92.66% | 197757 |
| NGS40 | T | - | sputum | *Mycobacterium ahvazicum* | 99.5 | 91.14% | 279661 |
| NGS26 | T | - | sputum | *Mycobacterium heidelbergense* | 85.57 | 92.93% | 708449 |
| NGS63 | H | - | sputum | *Mycobacterium alsense* | 89.71 | 92.46% | 699453 |
| NGS57 | H | - | sputum | *Mycobacterium alsense* | 88.96 | 93.31% | 369156 |

Supplementary Figure 1. Annual species distribution of probable NTM pulmonary disease across the three NTUH centers in Taiwan, 2019–2024.
